# Description of a new *Ornithodoros* (*Pavlovskyella*) (Ixodida: Argasidae) tick species from Pakistan

**DOI:** 10.1017/S0031182024000982

**Published:** 2024-08

**Authors:** Abid Ali, Mehran Khan, Muhammad Numan, Abdulaziz Alouffi, Mashal M. Almutairi, Ronel Pienaar, Minique H. de Castro, Lidia Chitimia-Dobler, Sebastián Muñoz-Leal, Ben J. Mans

**Affiliations:** 1 Department of Zoology, Abdul Wali Khan University Mardan, Khyber Pakhtunkhwa, Pakistan; 2 Infectious diseases, King Abdulaziz City for Science and Technology, Riyadh 12354, Saudi Arabia; 3 Department of Pharmacology and Toxicology, College of Pharmacy, King Saud University, Riyadh 11451, Saudi Arabia; 4 Epidemiology, Parasites and Vectors, Agricultural Research Council-Onderstepoort Veterinary Research, Onderstepoort 0110, South Africa; 5 Department of Zoology and Entomology, University of the Free State, P.O. Box 339, Bloemfontein 9300, South Africa; 6 The Biotechnology Platform, Agricultural Research Council-Biotechnology Platform, Onderstepoort 0110, South Africa; 7 Infection and Pandemic Research, Fraunhofer Institute of Immunology, Penzberg, Germany; 8 Experimental Parasitology, Department of Veterinary Sciences, Faculty of Veterinary Medicine, Ludwig-Maximilians-Universität, LMU, Munich, Germany; 9 Departamento de Ciencia Animal, Facultad de Ciencias Veterinarias, Universidad de Concepción, Chillán, Ñuble, Chile; 10 Department of Life and Consumer Sciences, University of South Africa, Private Bag X6, Roodepoort, Florida 1710, South Africa

**Keywords:** Argasidae, mitogenome, *Ornithodoros*, Pakistan, *Pavlovskyella*

## Abstract

The genus *Ornithodoros* is notably diverse within the family Argasidae, comprising approximately 134 species distributed among 4 subgenera, 1 of which is the subgenus *Pavlovskyella*. In an earlier study, we identified distinct soft ticks as *Ornithodoros* (*Pavlovskyella*) sp., which were collected from animal shelters in Khyber Pakhtunkhwa, Pakistan. Providing additional collections from that same locality and a comprehensive analysis involving detailed morphological and mitogenome-based comparisons with closely related species, this study formally designates a novel species for these specimens. Adults and late-instar nymphs of the new species display a dorsoventral groove, small cheeks not covering the capitulum, 5 small even humps on tarsus I and a transverse postanal groove intersecting the median postanal groove perpendicularly. It also lacks a tuft of setae on the ventral surface of the hood which separates the novel species from *Ornithodoros papillipes*. Ventral chaetotaxy of tarsus IV indicates 4–7 setal pairs in nymphs and 5–7 pairs in adults that separate the new species from *Ornithodoros tholozani* sensu stricto and *Ornithodoros crossi*, 2 morphologically closely related species that occur in geographical proximity. Phylogenetic analyses of the full-length mitochondrial genome and the 18S and 28S ribosomal RNA genes, combined with pairwise nucleotide comparisons of *cox1*, *cox2*, *atp8*, *atp6*, *cox3*, *nad3*, *nad5*, *nad4*, *nad4L*, *nad6*, *cytb*, *nad1*, *nad2*, 12S rDNA, 16S rDNA, 18S rDNA and 28S rDNA further support that the new species belongs to the *Pavlovskyella* subgenus, clustering with *O. tholozani*, *Ornithodoros verrucosus* and *Ornithodoros tartakovskyi*.

## Graphical abstract

**Figure:**
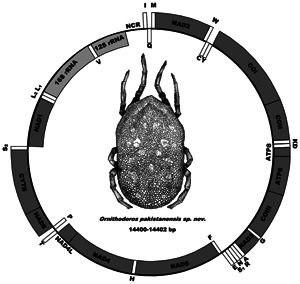

## Introduction

The Argasidae family consists of soft-bodied ticks that parasitize semi-terrestrial and terrestrial vertebrate hosts (Guglielmone *et al*., 2010; Manzano-Román *et al*., 2012). Soft ticks have a global distribution and are typically found in microclimates near their hosts (Vial, 2009; Dietrich *et al*., 2011; Estrada-Peña *et al*., 2017; Kleinerman and Baneth, 2017). They primarily feed at night for short periods but can endure extended periods without hosts (Estrada-Peña *et al*., 2017; Kleinerman and Baneth, 2017). They possess a ventrally located gnathostoma and lack a chitinous dorsal scutum (Vial, 2009; Estrada-Peña *et al*., 2017). Argasidae includes approximately 218 tick species in 15 genera, 1 of which is the genus *Ornithodoros* (Guglielmone *et al*., 2010; Mans *et al*., 2019; Mans, 2023).

*Ornithodoros* ticks are distinguished from other soft ticks by the absence of a ‘sutural line’ at the border between their dorsal and ventral surfaces (Estrada-Peña *et al*. 2010, 2017; Mans, 2023). The genus *Ornithodoros* is diverse, encompassing around 134 species distributed among 4 subgenera (Estrada-Peña *et al*., 2010, 2017; Muñoz-Leal *et al*., 2017; Mans *et al*., 2019, 2021; Dantas-Torres and Otranto, 2022). One of these subgenera is the *Pavlovskyella* (Supplementary Table 1), characterized by a combination of characters in adults: transverse postanal groove intersected by the median postanal groove, absence of large cheeks covering the capitulum, presence of dorsoventral groove and humps in tarsus I (Clifford *et al*., 1964; Filippova, 1966; Pospelova-Shtrom, 1969; Estrada Peña *et al*., 2010, 2017; Muñoz-Leal *et al*., 2017; Mans *et al*., 2019; 2021).

*Ornithodoros tholozani* is the type species of the subgenus and has a wide geographic distribution (Desportes and Campana, 1946). Three subspecies of *O. tholozani* have been proposed: *O. tholozani* sensu stricto (Iran, Iraq), *O. tholozani pavlovskyi* (Central Asia) and *O. tholozani crossi* (India) (Desportes and Campana, 1946). The latter subspecies, originally described as *Ornithodoros crossi*, was collected in Punjab, India (Brumpt, 1922), near the border with Pakistan. Although Sapre (1944) redescribed *O. crossi* using specimens collected in the type locality, he was unable to examine the type series that was lost. Additionally, the validity of this species had been questioned earlier by Leeson (1953). However, Sapre's redescription of *O. crossi* clearly indicates that *O. tholozani crossi* is not a synonym because of marked differences in the chaetotaxy of tarsus IV. Therefore *O. tholozani crossi* sensu Desportes & Campana should neither be considered a synonym of *O. crossi* nor of any other closely related species. We consider *O. crossi* a *nomen nudum* because the type series is missing. Noteworthily, *O. tholozani* was considered a synonym of *Ornithodoros papillipes* by the North American group of taxonomists (Hoogstraal, 1985). We consider *O. papillipes* valid because it has a denticulated tuft of setae arising from the hood, in front of the camerostome (Pavlovsky, 1930), and this character is absent in *O. tholozani* (Sapre, 1944). *Ornithodoros* species exhibit a lack of critical morphological features, which has historically led to confusion in species identification, emphasizing the necessity for molecular-based identification methods.

There are notable disagreements regarding the systematic classification of argasid ticks among 4 primary schemes: the Soviet scheme (Pospelova-Shtrom, 1946; Filippova, 1966), the American scheme (Clifford *et al*., 1964; Hoogstraal, and Kohls, 1966; Hoogstraal, 1985), the French scheme (Camicas and Morel, 1977; Camicas *et al*., 1998) and the morphological cladistic scheme (Klompen and Oliver, 1993). A more advanced classification scheme has recently been established based on mitochondrial genomes (Mans *et al*., 2012, 2019, 2021), which is the one adopted in this article. Additionally, studies have indicated that the genus *Ornithodoros*, along with some of its subgenera, including *Pavlovskyella*, are paraphyletic (Labruna *et al*., 2008; Barros-Battesti *et al*., 2011; Mans *et al*., 2019, 2021; Muñoz-Leal *et al*., 2023). The controversial phylogenetic relationships among argasid ticks could be addressed through mitogenomic and nuclear 18S–28S rDNA-based analysis of both the established and newly discovered argasid tick species.

The soft tick fauna of Pakistan has received limited attention. Given its zoogeographical location, which falls between the Palearctic and Oriental regions, Pakistan is expected to host a diverse range of soft tick species. Approximately, 9 soft tick species have been reported in this country, including 5 species (*Alveonasus lahorensis*, *Argas* sp. ‘rousetti’, *Argas persicus*, *Carios vespertilionis* and *Ornithodoros* sp.) identified through molecular analysis and 4 species identified based on morphological characteristics (*Argas abdussalami*, *O. papillipes*, *Argas reflexus* and *O. tholozani*) (Rao and Kalra, 1949; Doss *et al*., 1978; Hoogstraal, 1985; Karim *et al*., 2017; Zahid *et al*., 2021, 2023; Ali *et al*., 2022; Mans *et al*., 2024). The objective of this study was to thoroughly investigate the status of the previously undetermined *Ornithodoros* sp. (Ali *et al*., 2022) through detailed morphological and molecular analyses.

## Materials and methods

### Tick specimens and research area

**Figure 1.**
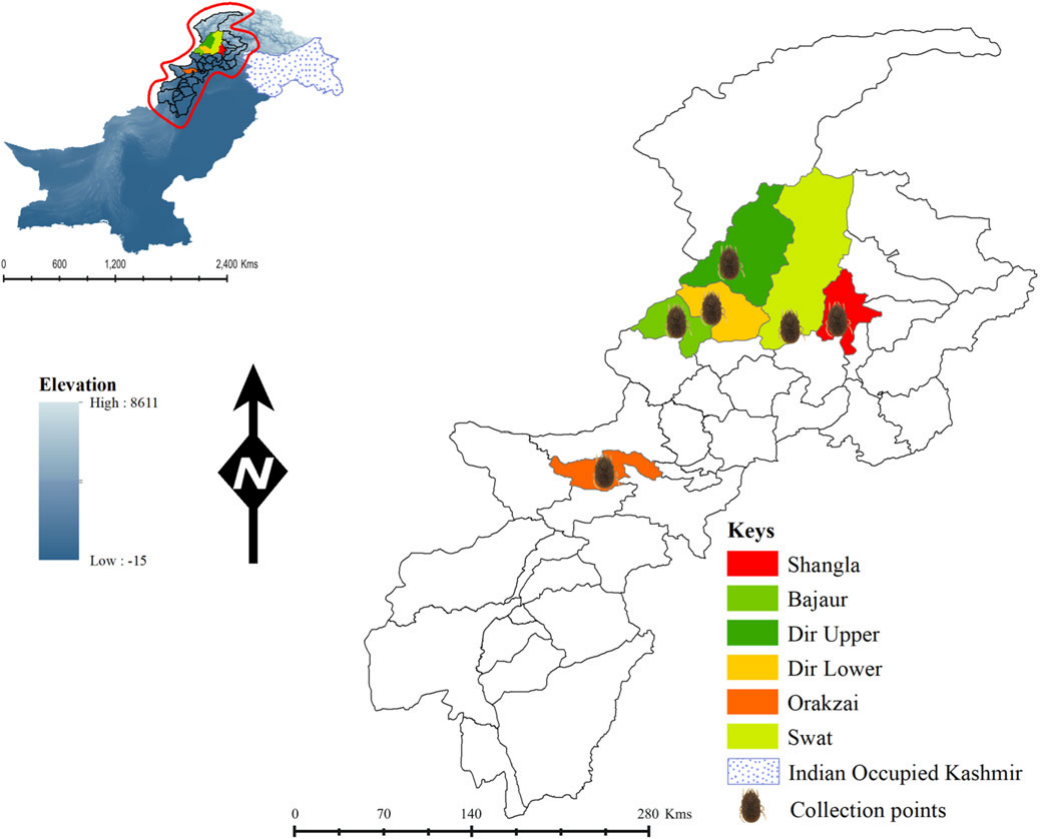
Map of Khyber Pakhtunkhwa province, Pakistan, showing the locations where *Ornithodoros* ticks were collected for this study.

*Ornithodoros* specimens for this study were obtained from previously reported specimens from 5 districts of Khyber Pakhtunkhwa, Pakistan: Shangla (34°47′19.9″N, 72°41′30.6″E), Bajaur (34°46′04.7″N, 71°29′49.1″E), Dir Upper (35°11′37.7″N, 71°54′17.2″E), Dir Lower (34°52′25.0″N, 71°46′49.2″E) and Orakzai (33°41′13.0″N, 70°59′48.7″E) (Ali *et al*., 2022). Additionally, a new collection from these districts was made, with the addition of specimens from the new district Swat (34°44′18.7″N, 72°20′50.6″E). These districts, situated in the northern province of Pakistan, are characterized by their mountainous landscapes, with elevations ranging from approximately 1500 to 3500 m. They experience a temperate climate, with cold winters and moderate summers. The environmental conditions support agriculture and forestry. The global positioning system or Google Maps (https://www.google.com/maps/) was used to determine the geographic coordinates of the collection sites, and ArcGIS v. 10.3.1 was used to design the study map (Fig. 1).

### Specimen collection and preservation

The *Ornithodoros* ticks were collected during 2019 and 2023 from cracks, crevices, burrows and debris in animal shelters (that housed small or large ruminants) throughout 6 monitoring locations/districts: Shangla, Bajaur, Dir Upper, Dir Lower, Orakzai and Swat in KP, Pakistan. The *Ornithodoros* specimens were collected in 15 mL Falcon tubes, and sent to the Department of Zoology, Abdul Wali Khan University, Mardan, Garden Campus, KP, Pakistan. To remove any contaminants from the body surface, the tick specimens were washed in distilled water and then with 70% ethanol. They were subsequently preserved in 100% absolute ethanol in Eppendorf tubes for further molecular analysis.

### Morphological analysis

The collected *Ornithodoros* specimens were identified morphologically using a stereo-zoom microscope (StereoBlue-euromex SB.1302-1, Arnhem, Netherlands) using the taxonomic key of Filippova (1966), because it includes the species of *Ornithodoros* occurring in Pakistan and other geographically related species. Late-instar nymphs and adult specimens were prepared for scanning electron microscopy (SEM). Briefly, specimens were sonicated in a solution of dish soap for 30 min, and dehydrated in a 70–100% ethanol battery to undergo critical point drying and metallization. Micrographs were captured with a JEOL JMS-5900 and a Hitachi SU35000 scanning electron microscope. The nomenclature of this study followed Cooley and Kohls (1944), Filippova (1966) and Muñoz-Leal *et al*. (2020). Specimens were deposited in the following tick collections: Colección Chilena de Garrapatas ‘Daniel González-Acuña’ (CCG) and the United States National Tick Collection (USNTC).

### Genomic DNA isolation and next-generation sequencing

DNA was extracted from *Ornithodoros* specimens using the QIAamp DNA Blood Mini Kit (Qiagen, Hilden, Germany) according to the manufacturer's instructions. NanoDrop (Nano-Q, Optizen, Daejeon, South Korea) was used to quantify the genomic DNA, which was then stored for further examinations at −20°C. Genomic DNA was processed using the MGIEasy Universal DNA Library Prep kit (MGI, Shenzhen, China) and sequenced on the MGI DNBSEQ-G400 sequencing instrument using the PE150 (paired-end 2 × 150 bp) format (Agricultural Research Council-Biotechnology Platform, South Africa) to obtain ~10 Gb of data per sample. The data quality and assembly protocol are similar to that obtained using Illumina paired-end short read sequencing previously used for the assembly of tick mitochondrial genomes (Mans *et al*., 2015, 2019, 2021). Specimens prepped for sequencing included Shangla, Bajaur and Dir Lower.

### Next-generation sequence assembly and evolutionary analyses

Paired-end sequence data were quality trimmed (0.001 quality limit) and MGI adapters removed using CLC Genomics Workbench v. 20.1 software (Qiagen Digital Insights, Aarhus, Denmark). Standard assembly parameters (mismatch cost-2, insertion cost-3, deletion cost-3, length fraction-0.9, similarity-0.9, minimum contig length-200 and automatic bubble size) were used and assembly performed using a kmer size of 49 in CLC Genomics Workbench v. 20.1 software (Qiagen Digital Insights). Contigs were identified as mitochondrial, 18S or 28S rDNA using BLASTn analysis (Altschul *et al*., 1990). Final contigs were obtained by mapping data back to the contigs using CLC Genomics Workbench v. 20.1 (mismatch cost-2, insertion cost-3, deletion cost-3, length fraction-0.5 and similarity-0.9), to obtain consensus sequences and final coverage values. The mitochondrial genome was annotated using the MITOS and ARWEN servers to identify tRNA genes (Laslett and Canbäck, 2008; Bernt *et al*., 2013). Protein coding genes were identified using the Expasy Translation Server (https://web.expasy.org/translate/) and BLASTp analysis (Altschul *et al*., 1990). Following BLAST, the maximum identity sequences were retrieved in the FASTA format from the NCBI for each gene used in the analysis including the *cox1* (cytochrome c oxidase subunit 1), *cox2* (cytochrome c oxidase subunit 2), *atp8* (ATP synthase F0 subunit 8), *atp6* (ATP synthase F0 subunit 6), *cox3* (cytochrome c oxidase subunit 3), *nad3* (NADH dehydrogenase subunit 3), *nad5* (NADH dehydrogenase subunit 5), *nad4* (NADH dehydrogenase subunit 4), *nad4L* (NADH dehydrogenase subunit 4L), *nad6* (NADH dehydrogenase subunit 6), *cytb* (cytochrome b), *nad1* (NADH dehydrogenase subunit 1), *nad2* (NADH dehydrogenase subunit 2), 12S rDNA (12S ribosomal DNA), 16S rDNA (16S ribosomal DNA), 18S rDNA (18S ribosomal DNA) and 28S rDNA (28S ribosomal DNA) sequences. The BioEdit Sequence Alignment Editor v. 7.0.5 was used for the alignment of the acquired sequences with the downloaded sequences using ClustalW multiple alignments (Hall *et al*., 2011). The obtained coding sequences were aligned by using the integrated MUSCLE algorithm (Edgar, 2004). The alignments of DNA (12S–16S rDNA and 18S–28S rDNA) and amino acid (*cox1*, *cox2*, *atp8*, *atp6*, *cox3*, *nad3*, *nad5*, *nad4*, *nad4L*, *nad6*, *cytb*, *nad1* and *nad2*) sequences were subjected to Molecular Evolutionary Genetics Analysis (MEGA-11) (Tamura *et al*., 2021) for concatenation into datasets. The concatenated alignment files were exported in the FASTA format and converted to the RELAXPHYLIP format *via* NCL converter v. 2.1 tool (Lewis, 2003), in the Cyberinfrastructure for Phylogenetic Research (CIPRES) Science Gateway v. 3.3 (CIPRES; https://www.phylo.org/portal2/) (Miller *et al*., 2010). The maximum-likelihood phylogenetic trees were constructed through the IQ-Tree v. 2.2.2.7 tool run on XSEDE in CIPRES (Miller *et al*., 2010) using default options with 1000 ULTRAFAST bootstrap replicates (Hoang *et al*., 2018). The output tree file was visualized by the FigTree v. 1.4.4 tool (Rambaut, 2018). The concatenated datasets were also subjected to MEGA-11, using the maximum composite likelihood model (Tamura *et al*., 2004), to determine pairwise genetic distances. The number of base substitutions per site between pairwise gene sequences was determined. These analyses involved 46, 48, 41 and 32 sequences for protein coding amino acid sequences, protein coding nucleotide sequences, 12S–16S rDNA and 18S–28S rDNA sequences, respectively.

## Results

### Morphological description of *Ornithodoros pakistanensis* sp. nov.

#### **Order:** Ixodida Leach, 1815 **Family:** Argasidae Canestrini, 1890 **Genus:** *Ornithodoros* Koch, 1837, 1844 *Ornithodoros pakistanensis* sp. nov. Ali, Chitimia-Dobler, Muñoz-Leal & Mans

**Type locality**: Khyber Pakhtunkhwa (KP), Pakistan.

**Type specimens:** Holotype: 1 female in ethanol (USNTC, in progress). Allotype: 1 male in ethanol at USNTC, same accession number. Paratypes: 5 males, 14 females, 46 nymphs in ethanol (CCG-86), 11 males, 9 females and 24 nymphs in ethanol at the Gertrud Theiler Tick Museum (ARC-OVR) and 202 males, 277 females, 207 nymphs in ethanol at the Department of Zoology, Abdul Wali Khan University, Mardan, KP, Pakistan.

**Etymology**: The species epitet ‘pakistanensis’ is in allusion to the geographical origin of this newly discovered species.

**Hosts**: Collected in cracks, crevices, burrows and debris of animal shelters that housed small or large ruminants (goats, sheep, cattle, buffaloes) and equids.

**Known infectious agents**: *Rickettsia* sp. belong to the limoniae group (Ali *et al*., 2022).

**Disease relationships**: Unknown.

**Zoobank registration number**: LSID: 0FC991C3-6B96-49D6-B23C-CB939D7BBFC7.

### Female (measurements based on 14 specimens, Fig. 2)

*Body:* Sub-oval, 5.775 ± 0.025 mm (5.750–5.800 mm) long, 3.340 ± 0.080 mm (3.260–3.420 mm) wide. Lateral margins subparallel, curving posteriorly, converging to a round apex anteriorly.

**Figure 2.**
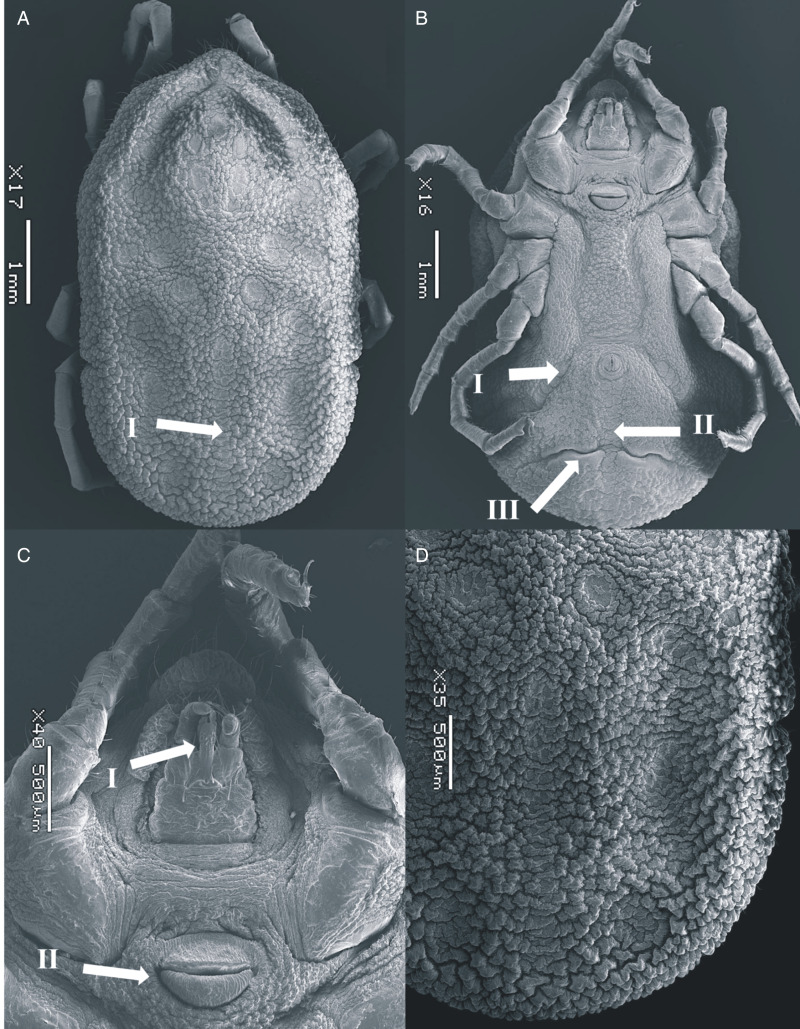
SEM of *Ornithodoros pakistanensis* female: (A) idiosoma dorsal view (I: posterior mammillae), (B) idiosoma ventral view (I: preanal grooves, II: medium postanal grooves and III: transverse postanal grooves), (C) ventral capitulum (I: hypostome and II: genital aperture) and (D) idiosoma dorsal/posterior mammillae collected in this study.

*Dorsum*: Covered by irregularly shaped mammillae, with a smooth surface top; tightly interlocked with irregular buttresses; larger in body margins; some with a small seta. Eyes absent. Dorsoventral groove present. Dorsal discs faint, pebbled with thick marginal ridges. Anteromedian disc present, anterior to centrolateral discs; anterolateral discs merged into a curved line forming a groove in the anterior margins of idiosome. Intermedian anterior and intermedian central discs merged; intermedian central and intermedian posterior discs not merged, separated diagonally. Median disc merged with the posteromedian file of discs, reaching the posterior margin of idiosome; posterolateral file begins posterior to median disc and intersected by mammillae.

*Venter*: Covered by small mammillae in the middle, larger mammillae in the posterior part. Discs present along preanal and median postanal grooves. Transverse postanal groove intersecting perpendicularly the median postanal groove slightly below the medial point from its origin in anal ring. Coxal folds devoid of glabrous patches of integument. Spiracular plates located between coxae III and IV. Genital area surrounded by a striated ring merging with the anterior lip that lacks a pore on its surface. Anterior and posterior lips similarly sized.

*Capitulum*: Located below a large hood. The hood devoid of setae. Cheeks small, not covering the capitulum. Basis capitulum rectangular, wrinkled, with 1 postpalpal and 1 posthypostomal pair of setae, similar in length. Three to 4 short basal setae laterally. Palpi elongated, provided with abundant setae. Palpal article I large, with a large ridge-like extension along its internal margin. Hypostome reaching the second palpal article in length; with crenulations at the tip and below the denticles. Two rows of 3–5 denticles present in the distal half, notched apically.

*Legs*: Without micromammillae. Coxae anteriorly elongated, with the anterior two-thirds sclerotized. Coxa I with a patch of micromammillae laterally. Coxae I–IV decreasing on size; coxae I and II separated, II–IV contiguous. Tarsus I with 5 small dorsal humps, equally protruding, tarsus II with 4; tarsus III and IV with an apical protuberance, without humps. Tarsus IV with 6–7 pairs of setae ventrally. Bifid claws and pulvillus present.

### Male (measurements based on 5 specimens, Fig. 3)

Body sub-oval 4.687 ± 0.042 mm (4.645–4.729 mm) long, 2.757 ± 0.052 mm (2.705–2.810 mm) wide, similar to female. Cheeks smaller than female, not covering the capitulum. Dorsum with mammillae slightly more separated than the female. Dorsal discs visible, topology as female. Hypostome short, reaching the first palpal article in length. Genital flap chiefly straight, with crenulations at the base. Legs similar to female.

**Figure 3.**
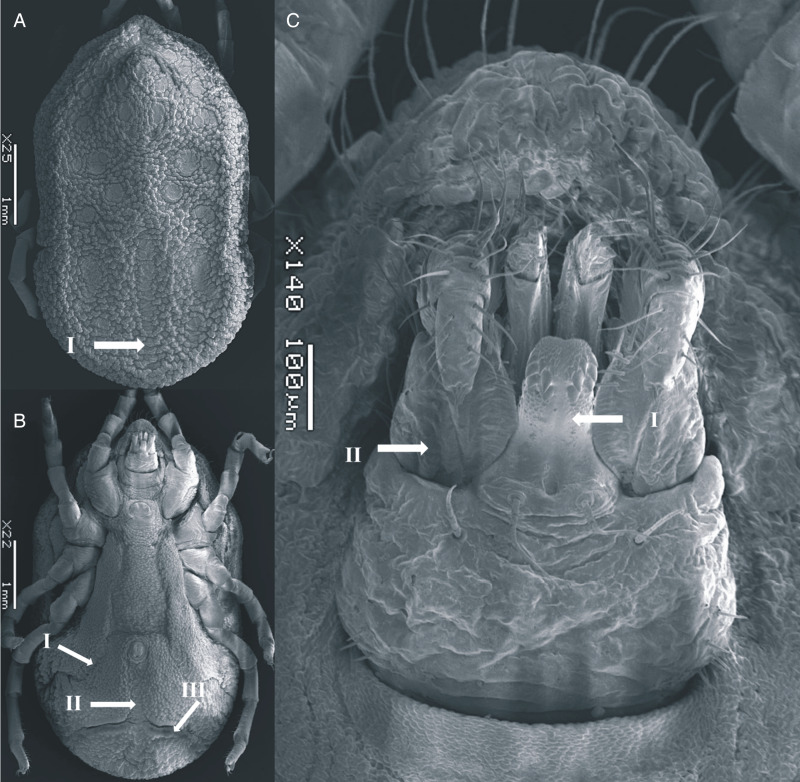
SEM of *O. pakistanensis* male: (A) idiosoma dorsal view (I: posterior mammillae), (B) idiosoma ventral view (I: preanal grooves, II: medium postanal grooves and III: transverse postanal grooves) and (C) ventral capitulum (I: hypostome and II: palps) collected in this study.

### Late-instar nymph (measurements based on 20 specimens, Fig. 4)

Body sub-oval 5.381 ± 0.168 mm (5.213–5.550 mm) long, 2.775 ± 0.025 mm (2.750–2.800 mm) wide. Morphologically similar to female but lacking genital apron. Tarsus IV provided with 4–6 pairs of ventral setae.

**Figure 4.**
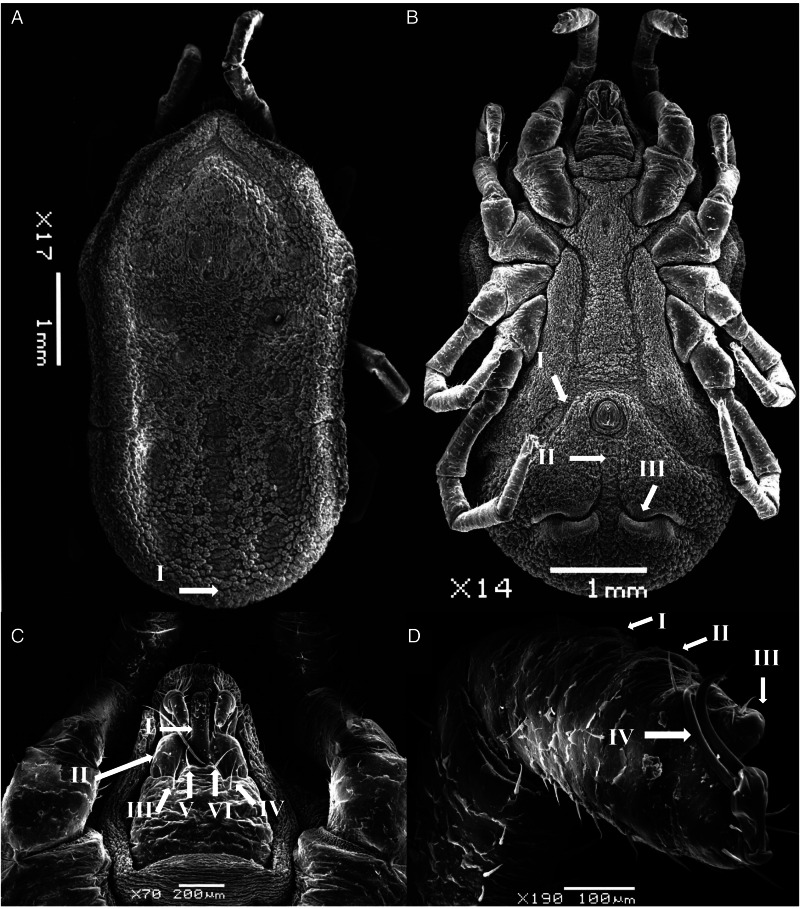
SEM of nymph stage for *O. pakistanensis*: (A) idiosoma dorsal view (I: posterior mammillae), (B) idiosoma ventral view (I: preanal grooves, II: medium postanal grooves and III: transverse postanal grooves), (C) ventral capitulum (I: hypostome, II: palps, III and IV: setae on capitulum, V and VI: postpalpal setae) and (D) legs (I–III: tarsus/metatarsus and IV: pair of spur) collected in this study.

### Genetic profiling and evolutionary analysis

**Figure 5.**
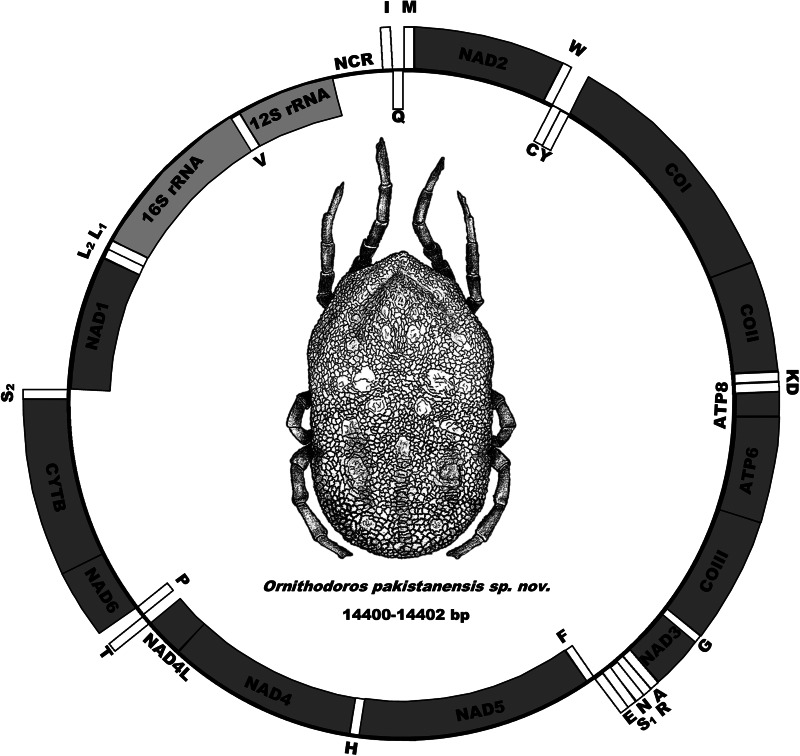
Mitochondrial genome arrangement of *O. pakistanensis* sp. nov. Arrangements of genes on the forward strand (outside, clockwise) and reverse strand (inside, anti-clockwise) is indicated.

The mitochondrial genome has the standard arrangement of 13 protein genes (*cox1*, *cox2*, *atp8*, *atp6*, *cox3*, *nad3*, *nad5*, *nad4*, *nad4L*, *nad6*, *cytb*, *nad1* and *nad2*), 2 ribosomal RNA genes (12S rDNA and 16S rDNA) and 22 transfer RNA genes as well as the gene structure observed for argasid ticks (Fig. 5) (Shao *et al*., 2004; Mans *et al*., 2012, 2019, 2021; Burger *et al*., 2014). Sequence alignment of the 3 mitochondrial genomes indicates pairwise identities of 99% and a mitochondrial genome size of 14 400–14 402 bp.

**Figure 6.**
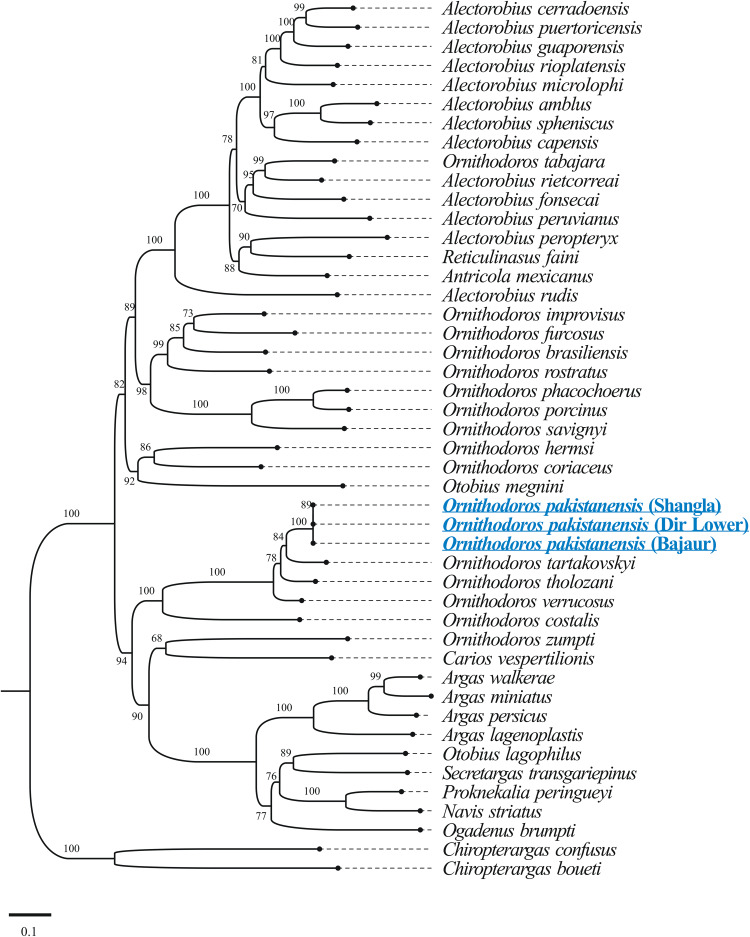
Maximum-likelihood tree for the concatenated 13 mitochondrial protein sequences (*cox1*, *cox2*, *atp8*, *atp6*, *cox3*, *nad3*, *nad5*, *nad4*, *nad4L*, *nad6*, *cytb*, *nad1* and *nad2*). The sequence of *Chiropterargas confusus* and *Chiropterargas boueti* was used as outgroup. The bootstrap values (1000 replicates) are shown at each node. The obtained sequences for *O. pakistanensis* are underlined and presented in blue.

The results of BLASTp for protein coding amino acid sequences of the mitogenome of *O. pakistanensis* sp. nov. are provided in Table 1. All concatenated protein coding amino acid sequences showed the lowest pairwise genetic distance of 0.128 in comparisons with *Ornithodoros verrucosus*, followed by 0.129 with *O. tholozani* and 0.133 with *Ornithodoros tartakovskyi* (Supplementary Table 2). Based on their maximum identities and concatenated phylogenetic analysis, the obtained protein coding amino acid sequences for *O. pakistanensis* sp. nov. clustered with species of the subgenus *Pavlovskyella*, namely *O. tholozani*, *O. verrucosus* and *O. tartakovskyi* (Fig. 6).

**Table 1.** Outcomes of BLASTp and BLASTn analyses for protein coding genes in the mitogenome *of Ornithodoros pakistanensis* sp. nov.

	Gene	BLAST analysis (per cent identity, query species, query sequence)
BLASTp	*cox1*	(97.65, *O. verrucosus*, YP_010535728), (97.26, *O. tartakovskyi*, YP_010535715), (96.87, *O. tholozani*, YP_009536334)
	*cox2*	(92, *O. tholozani*, YP_009536335), (91.11, *O. verrucosus*, YP_010535729), (90.22, *O. tartakovskyi*, YP_010535716)
	*atp8*	(80.77, *O. verrucosus*, YP_010535730), (78.85, *O. tartakovskyi*, YP_010535717), (78.85, *O. tholozani*, YP_009536336)
	atp6	(89.19, *O. tartakovskyi*, YP_010535718), (87.84, *O. verrucosus*, YP_010535731), (85.59, *O. tholozani*, YP_009536337)
	cox3	(93.82, *O. tholozani*, YP_009536338), (91.51, *O. verrucosus*, YP_010535732), (91.51, *O. tartakovskyi*, YP_010535719)
	nad3	(85.71, *O. tholozani,* YP_009536339), (84.82, *O. verrucosus,* YP_010535733), (83.04, *O. tartakovskyi,* YP_010535720)
	nad5	(87.91, *O. verrucosus*, YP_010535734), (87.91, *O. tholozani*, YP_009536340), (85.20, *O. tartakovskyi*, YP_010535721)
	*nad4*	(87.73, *O. verrucosus*, YP_010535735), (87.05, *O. tholozani*, YP_009536341), (80.37, *O. tartakovskyi*, YP_010535722)
	nad4L	(89.01, *O. verrucosus*, P_010535736), (85.71, *O. tholozani*, YP_009536342), (83.52, *O. tartakovskyi*, YP_010535723)
	nad6	(83.08, *O. tartakovskyi*, YP_010535724), (82.76, *O. tholozani*, YP_009536343), (81.38, *O. verrucosus*, YP_010535737)
	*cytb*	(92.66, *O. tartakovskyi*, YP_010535725), (92.12, *O. verrucosus*, YP_010535738), (90.76, *O. tholozani*, YP_009536344)
	*nad1*	(87.70, *O. verrucosus*, YP_010535739), (87.33, *O. tartakovskyi*, YP_010535726), (87.26, *O. tholozani*, YP_009536345)
	*nad2*	(80.69, *O. verrucosus*, YP_010535727), (79.06, *O. tartakovskyi*, YP_010535714), (78.82, *O. tholozani*, YP_009536333)
BLASTn	*cox1*	(87.25, *O. verrucosus*, ON800889.1 and NC_067925.1), (86.21, *O. tholozani*, NC_039830.1), (85.18, *O. tartakovskyi*, ON800883.1, NC_067924.1)
	*cox2*	(88.04, *O. verrucosus*, ON800889.1 and NC_067925.1), (87.04, *O. tholozani*, NC_039830.1), (83.80, *O. tartakovskyi*, ON800883.1, NC_067924.1)
	*atp8*	(85.53, *O. verrucosus*, ON800889.1 and NC_067925.1), (85.44, *O. tartakovskyi*, ON800883.1, NC_067924.1), (83.54, *O. tholozani*, NC_039830.1)
	atp6	(83.73, *O. verrucosus*, ON800889.1 and NC_067925.1), (81.16, *O. tholozani*, NC_039830.1), (80.71, *O. tartakovskyi*, ON800883.1, NC_067924.1)
	cox3	(86.56, *O. verrucosus*, ON800889.1 and NC_067925.1), (87.55, *O. tholozani*, NC_039830.1), (84.36, *O. tartakovskyi*, ON800883.1, NC_067924.1)
	nad3	(83.58, *O. verrucosus*, ON800889.1 and NC_067925.1), (80.82, *O. tholozani*, NC_039830.1), (79.06, *O. tartakovskyi*, ON800883.1, NC_067924.1)
	nad5	(86.26, *O. verrucosus*, ON800889.1 and NC_067925.1), (84.44, *O. tholozani*, NC_039830.1), (83.77, *O. tartakovskyi*, ON800883.1, NC_067924.1)
	*nad4*	(86.10, *O. verrucosus*, ON800889.1 and NC_067925.1), (84.35, *O. tholozani*, NC_039830.1), (80.64, *O. tartakovskyi*, ON800883.1, NC_067924.1)
	nad4L	(88.13, *O. verrucosus*, ON800889.1 and NC_067925.1), (84.89, *O. tholozani*, NC_039830.1), (82.73, *O. tartakovskyi*, ON800883.1, NC_067924.1)
	nad6	(85.19, *O. verrucosus*, ON800889.1 and NC_067925.1), (83.06, *O. tholozani*, NC_039830.1), (81.55, *O. tartakovskyi*, ON800883.1, NC_067924.1)
	*cytb*	(85.33, *O. verrucosus*, ON800889.1 and NC_067925.1), (84.22, *O. tholozani*, NC_039830.1), (83.27, *O. tartakovskyi*, ON800883.1, NC_067924.1)
	*nad1*	(88.13, *O. verrucosus*, ON800889.1 and NC_067925.1), (85.36, *O. tartakovskyi*, ON800883.1, NC_067924.1), (84.00, *O. tholozani*, NC_039830.1)
	*nad2*	(81.57, *O. verrucosus*, ON800889.1 and NC_067925.1), (79.13, *O. tholozani*, NC_039830.1), (77.64, *O. tartakovskyi*, ON800883.1, NC_067924.1)

*O. verrucosus* (Ukraine), *O. tholozani* (Israel) and *O. tartakovskyi* (Rocky Mountain Laboratory colony).

**Figure 7.**
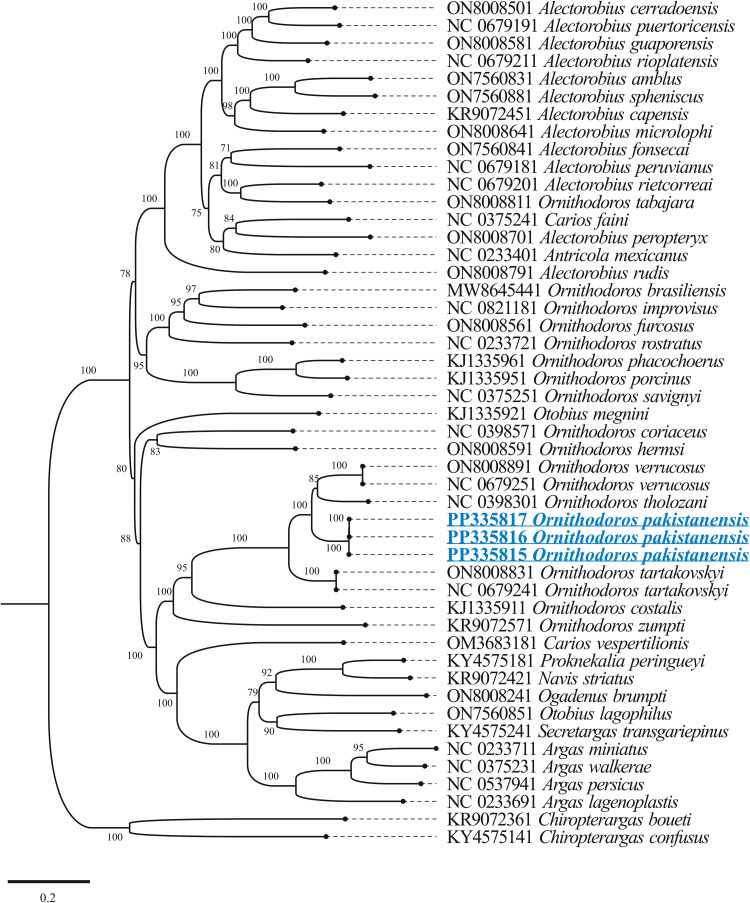
Maximum-likelihood phylogenetic tree 13 concatenated protein coding nucleotide sequences (*cox1*, *cox2*, *atp8*, *atp6*, *cox3*, *nad3*, *nad5*, *nad4*, *nad4L*, *nad6*, *cytb*, *nad1* and *nad2*). *Chiropterargas confusus* and *C. boueti* were used as an outgroup. The bootstrap values (1000 replicates) are shown at each node. The obtained sequences for *O. pakistanensis* are underlined and coloured in blue.

The outcomes of BLASTp for protein coding nucleotide sequences of the mitogenome of *O. pakistanensis* sp. nov. are presented in Table 1. All concatenated protein coding nucleotide sequences showed lowest pairwise genetic distance of 0.306 in comparisons with *O. verrucosus*, followed by 0.345 with *O. tholozani* and 0.393 with *O. tartakovskyi* (Supplementary Table 3). In the concatenated phylogenetic tree based on protein coding nucleotide sequences, *O. pakistanensis* sp. nov. clustered with *O. tholozani*, *O. verrucosus* and *O. tartakovskyi* of the subgenus *Pavlovskyella* (Fig. 7).

**Figure 8.**
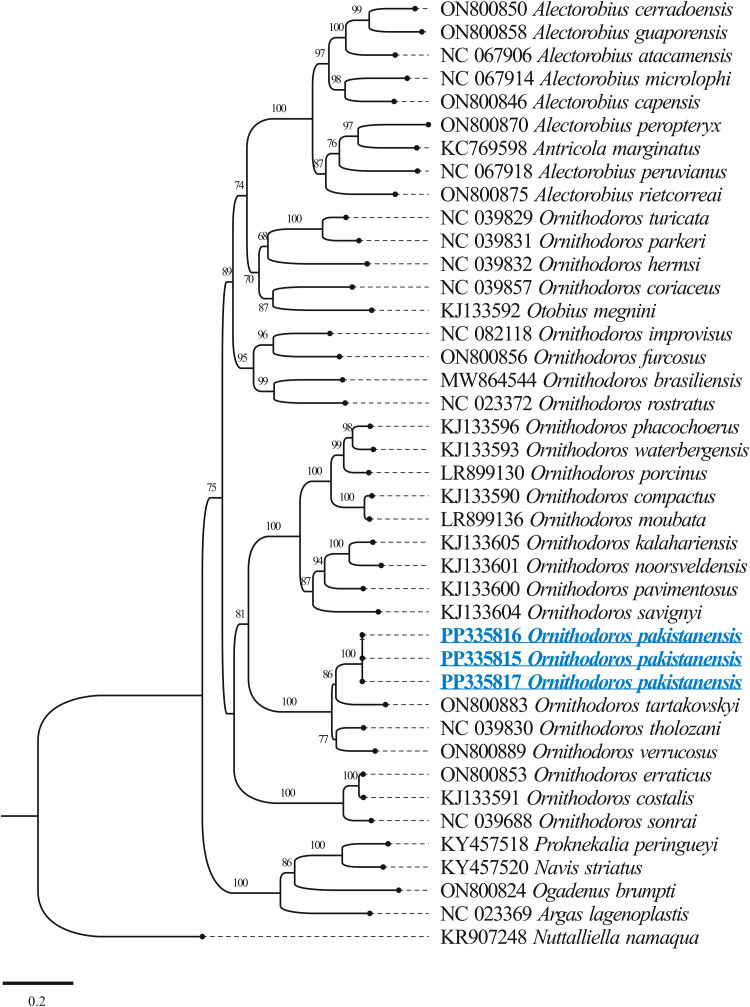
Maximum-likelihood phylogenetic tree based on 12S (684 bp)–16S (1022 bp) rDNA sequences The sequence of *Nuttalliella namaqua* was used as an outgroup. The bootstrap values (1000) are shown at each node. The obtained sequences for the present study are underlined and coloured in blue.

The BLASTn analysis of the obtained 12S rDNA sequence (684 bp) for *O. pakistanensis* sp. nov. showed 88.47% maximum identity with *O. tartakovskyi* (ON800883) followed by 87.65% with *O. verrucosus* (ON800889), and 87.54% with *O. tholozani* (NC039830). The obtained 16S rDNA sequence (1022 bp) of *O. pakistanensis* sp. nov. showed 88.69% maximum identity with *O. tholozani* (NC039830) followed by 86.91% with *O. verrucosus* (ON800889), and 85.27% with *O. tartakovskyi* (ON800883). The 12S–16S rDNA sequences showed the lowest 0.125 pairwise genetic distance in comparisons with *O. tholozani* (NC039830) followed by 0.144 with *O. verrucosus* (ON800889) and 0.151 with *O. tartakovskyi* (ON800883) (Supplementary Table 4). Based on their concatenated phylogenetic tree, the 12S–16S rDNA sequences were clustered with the *O. tartakovskyi*, *O. tholozani* and *O. verrucosus* reported from Rocky Mountain Laboratory colony, Israel and Ukraine, respectively (Fig. 8).

**Figure 9.**
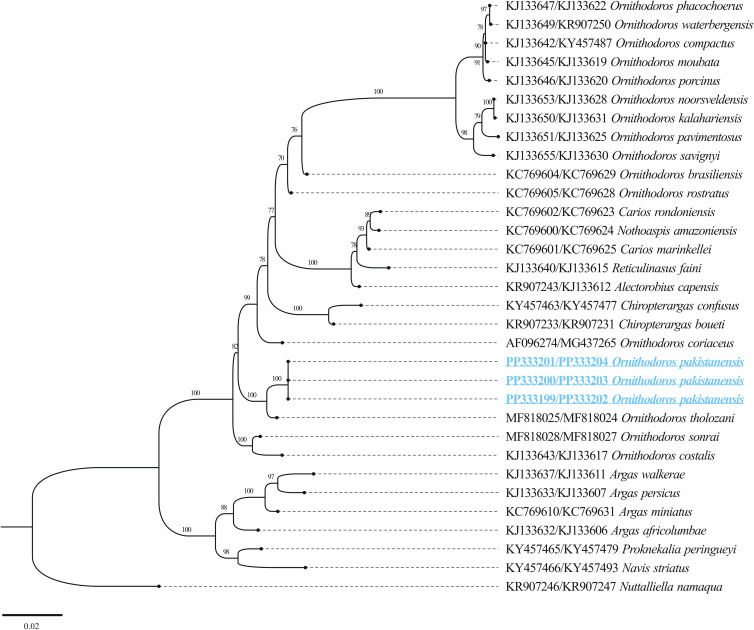
Maximum-likelihood phylogenetic tree based on the concatenated 18S (1539 bp)–28S (1377 bp) rDNA sequences. The sequence of *N. namaqua* was used as an outgroup. The bootstrap values (1000 replicates) are shown at each node. The obtained sequences for the present study are underlined and coloured in blue.

The BLASTn analysis of the obtained 18S rDNA sequence (1539 bp) and 28S rDNA sequence (1377 bp) for *O. pakistanensis* sp. nov. showed 99.09% (MF818025) and 99.27% (MF818024), maximum identity with *O. tholozani*, respectively. Matrix of evolutionary differences on pairwise comparisons of the mitochondrial 18S–28S rDNA sequences revealed small genetic divergence (0.009) with *O. tholozani* (MF818025/MF818024), followed by 0.022 with *Ornithodoros sonrai* (MF818028/MF818027) (Supplementary Table 5). Based on their concatenated phylogenetic tree, the 18S–28S rDNA sequences were clustered with the *O. tholozani* sequence reported from Israel (Fig. 9).

The obtained full-length mitochondrial genome (PP335815, PP335816, PP335817), 18S rDNA (PP333199, PP333200, PP333201) and 28S rDNA (PP333202, PP333203, PP333204) sequences for *O. pakistanensis* sp. nov. were deposited in GenBank.

## Discussion

The morphological based identification of *Ornithodoros* spp. has caused some historical confusions (Bakkes *et al*., 2018). Conducting comprehensive morphological and molecular analyses across a broad geographic range is essential for the accurate identification of both the established and newly discovered *Ornithodoros* ticks. This approach not only contributes to a more precise understanding of systematics of the genus *Ornithodoros* but also facilitates an accurate assessment of the epidemiology of the infectious agents they transmit. Herein, *O. pakistanensis* sp. nov. was described as belonging to the subgenus *Pavlovskyella* based on morphology, the mitochondrial genome and the nuclear 18S–28S rDNA. This species demonstrated close morphological and molecular resemblances to species within the subgenus *Pavlovskyella,* such as *O. tholozani*, while maintaining distinct characteristics that validate it as a new or separate species.

### Morphological species relationship

Adults and late-instar nymphs of *O. pakistanensis* sp. nov. are similar to other representatives of the *Pavlovskyella* subgenus. However, the novel species can be separated from *O. papillipes* because it lacks a tuft of setae on the ventral surface of the hood (Pavlovsky, 1930). *Ornithodoros pakistanensis* sp. nov. separates from *Ornithodoros cholodkovskyi* because in the latter species the transverse postanal groove intersects the median postanal groove clearly before the median point from its origin in the anal ring (Pavlovsky, 1930). *Ornithodoros tartakovskyi* is also similar to *O. pakistanensis* sp. nov., but the transverse postanal groove of the former forms a sharp angle in its intersection with the median postanal groove (Filippova, 1966). Moreover, *O. tartakovskyi* has 3 bulky humps on tarsus I compared to 5 small humps in *O. pakistanensis* sp. nov. The novel species is also similar to *O. verrucosus*; however, in the latter species the third of 5 humps is larger and higher than the others (Filippova, 1966). In *O. pakistanensis* sp. nov. all humps on tarsus I are evenly protruding. The comparison of closely related *O. tholozani* subspecies and *O. crossi* with *O. pakistanensis* sp. nov. needs to take into account the ventral pairs of setae on tarsus IV (Table 2). However, the holotype and original description of *O. crossi* are missing (Leeson, 1953) and *O. crossi* is considered a *nomen nudum*. We therefore based our comparisons with the redescription of the species, which states (in drawings) that *O. crossi* has 2 pairs of ventral setae in tarsus IV (Sapre, 1944). Instead, *O. pakistanensis* sp. nov. has 4–7 pairs in nymphs and 5–7 pairs in adults. The comparison of the chaetotaxy of tarsus IV has been previously studied in *O. tholozani* from different localities and supported the designation of 3 subspecies; particularly, *O. tholozani crossi* was proposed for the specimens collected in Punjab, India (Descamps and Campana, 1946). Considering this character, and the geographical proximity where the collections were carried out in Pakistan, *O. tholozani crossi* should be now regarded as *Ornithodoros pakistanensis* sp. nov. (Table 2).

**Table 2.** Chaetotaxy of tarsus IV of *O. tholozani* subspecies sensu Descamps & Campana (1946) and *O. pakistanensis* sp. nov.

Locality	Species	4 + 3	4 + 4	4 + 5	5 + 5	5 + 6	5 + 7	6 + 6	6 + 7	7 + 7	7 + 8	8 + 8	8 + 9	9 + 9	9 + 10
Iran	*O. tholozani* sensu stricto	2N	4N, 2♂	8N, 1♂	7N, 2♂, 1♀	4N, 2♂, 3♀	1♀	1♂, 1♀	–	–	–	–	–	–	–
Iraq	*O. tholozani* sensu stricto	2N, 1♀	3♀	1♂, 3♀	2♂, 7♀	1♂, 1♀		1♀	–	–	–	–	–	–	–
Syria	*O. tholozani* sensu stricto	2N	1N, 1♂	–	–	1N	–	–	–						–
Ferghana Valley (Central Asia)	*O. tholozani* *pavlovskyi*	–	–	–	–	–	–	1♂, 1♀	1♂	1♂, 5♀	1♂, 3♀	1♂, 4♀	1♀	1♀	1♀
Tajikistan (Central Asia)	*O. tholozani* *pavlovskyi*	–	–	–	–	–		1N	1N	3N	–	–	–	–	
Punjab (India)	*Ornithodoros tholozani* *crossi*		–	3N	2N	1N	–	1N, 1♀	1N, 1♂, 1♀		1♀	–	–	–	
Pakistan (KP)	*O. pakistanensis* sp. nov.	–	–	1N	6N	10N, 1♂	–	27N, 5♂, 7♀	2N	3♀	–	–	–	–	

The number of setae is separated by ‘+’ because not all the setae were paired.

### Phylogenetic species relationship

Particular focus on mitochondrial genome sequences offers the potential to elucidate disputed phylogenetic relationships within soft ticks (Burger *et al*., 2014; Mans *et al*., 2019, 2021; Mohamed *et al*., 2022). Consequently, ticks identified based on their morphology in this study were subjected to molecular characterization through mitogenome sequencing. Utilizing molecular analysis of various genes, *O*. *pakistanensis* sp. nov. was determined to be most closely related to *O. tholozani* sensu stricto, *O. verrucosus* and *O. tartakovskyi*. In contrast to the minimal divergence observed in 18S rDNA and 28S rDNA (less than 1%), 12S rDNA, 16S rDNA and the mitogenome displayed a substantial divergence of more than 12%. The former indicates high levels of conservation between species for the nuclear rDNA genes, the latter confirms the distinct nature of different species (Labruna *et al*., 2008; Mans *et al*., 2019, 2021; Khan *et al*., 2022; Ali *et al*., 2024). In the phylogenetic analysis using these genes, *O*. *pakistanensis* sp. nov. clustered within a monophyletic clade alongside *O. tholozani*, either independently (based on 18S rDNA and 28S rDNA), or in combination with other species such as *O. verrucosus* and *O. tartakovskyi* (based on *cox1*, 12S rDNA and 16S rDNA). Although there may be a common ancestor for these species, as suggested by sharing the subgenus *Pavlovskyella* (Kneubehl *et al*., 2022; Carnero-Morán *et al*., 2023), they exhibit significant variations in morphology, host preference and habitat use.

### On the presence of *O. tholozani* in Pakistan

Climate is considered to have a significant influence, when compared to biological factors such as host availability and vegetation patterns, on the distribution of *Ornithodoros* ticks (Cumming, 2002; Vial *et al*., 2018). While confirming the climatic suitability of central Asia for the establishment of *Ornithodoros* ticks, Vial *et al*. (2018) were unable to confirm the climate suitability of Pakistan due to poor evidence of any *Ornithodoros* species in the country, particularly *O. tholozani* sensu stricto. However, the region meets essential climate criteria for *Ornithodoros* feeding activity, including a spring temperature surpassing 10°C, a 3-month summer temperature exceeding 20°C, annual precipitation ranging between 60 and 750 mm, dry seasons interspersed by small rain showers and availability of residual water from perennial rivers near habitats.

*Ornithodoros* spp. typically thrive in microclimates within arid regions, infesting single or multiple vertebrate hosts during nighttime (Hoogstraal, 1985; Oliver, 1989; Guglielmone *et al*., 2010). *Ornithodoros tholozani*, known for infestations in both domestic and wild animals, is widely distributed in the deserts and semi-desert regions of Asia (Parola and Raoult, 2001; Estrada-Peña *et al*., 2017). Without any substantial evidence, this species has been considered to exist in Pakistan (Vial *et al*., 2018). Herein, *O*. *pakistanensis* sp. nov., a morphologically closely related species, is reported in the northwestern mountainous terrain of Pakistan, which comprises a range of semi-arid to humid regions, characterized by significant vegetation (Ali *et al*., 2022; Khan *et al*., 2023; Tila *et al*., 2023). Moreover, based on specific collection sites, it is suggested that domestic hosts such as cattle and small ruminants are the hosts of *O. pakistanensis* sp. nov.

## Conclusion

This study described *O. pakistanensis* sp. nov., a new species from the northwestern part of Pakistan using morphological and molecular analyses. Given that the genus *Ornithodoros* and the subgenus *Pavlovskyella* are paraphyletic, this study adds a novel species that fits *Pavlovskyella* sensu stricto morphology and genetics, closely related to *O. tholozani*, the type species of the subgenus. *Ornithodoros pakistanensis* can be morphologically distinguished from *O. tholozani* by the presence of 4–7 setal pairs in tarsus IV ventral chaetotaxy in nymphs, and 5–7 pairs in adults. Due to the overlap in several aspects of these 2 tick species, including morphology and distribution, the reliability of certain previous reports describing *O. tholozani* in the region, especially in Pakistan, may be questionable.

## Supporting information

Ali et al. supplementary material 1 Ali et al. supplementary material

Ali et al. supplementary material 2 Ali et al. supplementary material

Ali et al. supplementary material 3 Ali et al. supplementary material

Ali et al. supplementary material 4 Ali et al. supplementary material

Ali et al. supplementary material 5 Ali et al. supplementary material

## Data Availability

All data in the study is available in the public databases as listed by their accession numbers.

## Supplementary material

The supplementary material for this article can be found at https://doi.org/10.1017/S0031182024000982
