# Supplementary material for: Description of a new Ornithodoros (Pavlovskyella) (Ixodida: Argasidae) tick species from Pakistan
Source: Parasitology. 2024 Oct 2;151(9):919–32. doi: 10.1017/S0031182024000982 (PMC11770527; doi:10.1017/S0031182024000982)
Supplement: Ali et al. supplementary material 5 — Ali et al. supplementary material [file S0031182024000982sup005.docx]

**Table S1: A list of the members of the sub-genus *Pavlovskyella*. Adapted from Mans et al., 2019.**
